# Rapid Assessment of Metabolomic Fingerprinting of Recycled Sunflower By-Products via DART-HRMS

**DOI:** 10.3390/molecules29174092

**Published:** 2024-08-29

**Authors:** Carmela Zacometti, Anna Lante, Miluska Cisneros, Andrea Massaro, Dasha Mihaylova, Vesela Chalova, Albert Krastanov, Hristo Kalaydzhiev, Giorgia Riuzzi, Alessandra Tata, Severino Segato

**Affiliations:** 1 Istituto Zooprofilattico Sperimentale delle Venezie, Experimental Chemistry Laboratory, Viale Fiume, 78, 36100 Vicenza, Italy; czacometti@izsvenezie.it (C.Z.); amassaro@izsvenezie.it (A.M.); atata@izsvenezie.it (A.T.); 2 Department of Agronomy, Food, Natural Resources, Animals, and Environment-DANAE, Padova University, Viale dell’Università, 16, 35020 Legnaro, Italy; miluskaalexandra.cisnerosyupanqui@phd.unipd.it; 3 Department of Biotechnology, University of Food Technologies, 26 Maritza Blvd., 4002 Plovdiv, Bulgaria; dashamihaylova@yahoo.com (D.M.); abtkrst@hotmail.com (A.K.); hristo.kalaydzhiev@yahoo.com (H.K.); 4 Department of Biochemistry and Molecular Biology, University of Food Technologies, 26 Maritza Blvd., 4002 Plovdiv, Bulgaria; veselachalova@gmail.com; 5 Department of Animal Medicine, Production and Health, Padova University, Viale dell’Università, 16, 35020 Legnaro, Italy; giorgia.riuzzi@studenti.unipd.it (G.R.); severino.segato@unipd.it (S.S.)

**Keywords:** sunflower by-product valorisation, ethanol-wash solutes, ultrasound assisted extraction, ambient ionisation, DART-HRMS, PLS-DA, polyphenols

## Abstract

To comply with a more circular and environmentally friendly European common agricultural policy, while also valorising sunflower by-products, an ultrasound assisted extraction (UAE) was tested to optimise ethanol-wash solutes (EWS). Furthermore, the capabilities of DART-HRMS as a rapid and cost-effective tool for determining the biochemical changes after valorisation of these defatted sunflower EWS were investigated. Three batches of EWS were doubly processed into optimised EWS (OEWS) samples, which were analysed via DART-HRMS. Then, the metabolic profiles were submitted to a univariate analysis followed by a partial least square discriminant analysis (PLS-DA) allowing the identification of the 15 most informative ions. The assessment of the metabolomic fingerprinting characterising EWS and OEWS resulted in an accurate and well-defined spatial clusterization based on the retrieved pool of informative ions. The outcomes highlighted a significantly higher relative abundance of phenolipid hydroxycinnamoyl-glyceric acid and a lower incidence of free fatty acids and diglycerides due to the ultrasound treatment. These resulting biochemical changes might turn OEWS into a natural antioxidant supplement useful for controlling lipid oxidation and to prolong the shelf-life of foods and feeds. A standardised processing leading to a selective concentration of the desirable bioactive compounds is also advisable.

## 1. Introduction

Among oilseed crops, sunflower (*Helianthus annuus* L.) is widespread all over the world, ranking fourth after palm, soybean, and rapeseed edible oils [1]. Indeed, sunflower oil is largely used as salad dressing or cooking oil in more than 70 countries and contributes approximately 12% towards the edible oil production globally [2]. A protein-rich meal residue remains after oil extraction, and it may pose economic and environmental constraints if such high-biodegradable and oxygen-demanding by-product is discarded from the sunflower industry as untreated waste [3]. According to the more environmentally friendly agenda of the European Union (EU) common agricultural policy (CAP), the plant oil sector should ensure its transition to a circular economy, aiming for a zero-waste production from all the different steps of seed processing. Thus, innovative solutions should be explored to obtain the maximum value from these protein-rich seed flours, which includes the extraction of many nutritional macroconstituents as well as bioactive compounds and their re-circulation as value-added ingredients for the production of foodstuffs [4,5] or nutritionally enriched human-edible foods [6,7]. Recent scientific evidence has demonstrated the bioactivity of this sunflower residual cake after oil extraction from whole seeds, confirming its nutritional and biological potential as a valuable source of both proteins [8] and polyphenols [9]. The principal phenolic constituents of sunflower seeds are mostly a pool of acids, including chlorogenic, caffeic, cinnamic, coumaric, ferulic, sinapic, and hydroxy-cinnamic as well as traces of vanillic, syringic, and hydroxy-benzoic acids [10]. However, the polyphenols found in sunflower meal have been noted to adversely affect the digestibility, functional properties, and taste of the protein isolates coming from this material [11].

To enhance the quality of extracted proteins and bioactive compounds, previous studies investigated the employment of an innovative method based on the washing of the defatted sunflower seed residuals with ethanol [12,13]. The resulting ethanol-washed fluids were collected, vacuum concentrated, and transformed into a novel product known as ethanol-wash solutes (EWS). The production of these EWS has already been suggested by some authors [13] as a pilot lab-scale experimental alternative useful strategy to recycle wastes from the sunflower supply chain, since it was repurposed into a novel product rich in carbohydrates, lipids, and valuable bioactive compounds, including phenols and flavonoids. Given the market request for sustainable production, this transformation emphasises its potential as a powerful source of essential nutrients and beneficial phytochemicals, and as an innovative, value-added protein-based ingredient. The extraction of these bioactive compounds can be carried out with conventional techniques; however, such methods often require high temperatures, long extraction times, and large amounts of solvents, which can degrade valuable compounds, undermining their biological functions. In contrast, green technologies, like ultrasound-assisted extraction (UAE), offer a more sustainable and efficient approach, preserving the nutritional and metabolic integrity of these beneficial compounds [14]. Furthermore, to come up with innovative solutions for the valorisation of food by-products and wastes, researchers must rapidly detect the impact of the developed technologies. Such technologies offer the possibility of biorefining and harmonising the organoleptic, nutritional, and metabolic properties of several plant by-products, turning them into high value ingredients for the production of foods enriched with natural sources of bioactive compounds for nutraceutical purposes [15]. For these reasons, the determination of the biochemical composition of defatted sunflower seed residuals is also crucial to reach an in-depth knowledge about what types of processes could be more suitable [16].

Indeed, the diversity and chemical complexity of miscellaneous analytes make their assessment in defatted residuals, as well as in chemically treated, washed, and physically treated resulting matrices, a great challenge for a zero-waste plant oil supply chain. In addition to wet chemistry analyses, emerging multi-analytical techniques can ensure a detailed picture of the composition of both by-products and their nutritionally enriched derivates, allowing the simultaneous characterisation of a large number of bioactive compounds potentially useful to enhance farmed animal or human health. A reliable assessment of the metabolomic fingerprint modifications occurring along the chemical and ultrasound-assisted steps of the processing of sunflower wastes into a final, targeted powdered ingredient can be powerful for speculating on the changes in the chemical, structural, and functional properties. In this chemometric context, non-targeted metabolomic profiling, enabled by ambient mass spectrometry (AMS) and advanced analytical tools, has significantly enhanced both by-product safety and the authentication of the adopted production process [17,18]. AMS involves the direct desorption and ionization of sample analytes under ambient conditions, often requiring minimal or no pre-treatment [19], and it enables a real-time chemical analysis, producing mass-to-charge (*m*/*z*) profiles. Recent advancements in AMS for authentication [20,21,22] and characterisation [23,24,25] of edible oils confirmed that this analytical technology provides detection readouts rapidly, significantly decreasing the time needed for analysis, and guaranteeing rapid and affordable identification of biomarkers while also ensuring compliance with food safety and quality. Among the multiple AMS techniques, the application of direct analysis in real-time high-resolution mass spectrometry (DART-HRMS) facilitates precise, rapid, and highly sensitive fingerprinting and biomarker analysis of wastes from animal and plant raw material and their processed food products, together with statistical analysis [26,27]. A recent study also highlighted the capability of DART-HRMS to detect metabolomic fingerprinting changes occurring in dairy by-products obtained from the microparticulation and fermentative processes of native whey recycling, a challenging assessment of the diversity among chemically complex fluids due to miscellaneous analytes [28]. Multivariate statistical analysis is typically utilised because of its adaptability and effectiveness at handling complex datasets, extracting changing metabolite profiles according to geographical origins and feeding systems [29], seasonal variations [30], and industrial processes [31].

This study aims to assess the use of DART-HRMS as a tool for rapid analysis of the metabolic fingerprinting of an end-up pilot lab-scale innovative optimised defatted recycled sunflower by-product, hereby named OEWS. Specifically, the biochemical comparison between the ethanol-wash solutes (EWS) and their derivates after an optimisation process (OEWS) based on ultrasound assisted extraction (UAE) was made. In detail, univariate analysis followed by partial least square discriminant analysis (PLS-DA) were applied within a modelling approach to assess the most significant biochemical changes occurring after UAE treatment of EWS.

## 2. Results and Discussion

### 2.1. DART-HRMS Spectra

**Figure 1:**
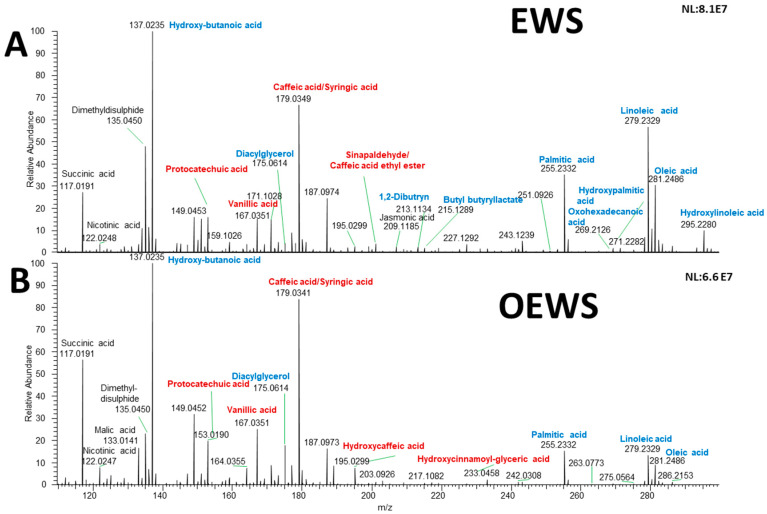
Representative DART-HRMS spectra of the ethanol-wash solutes, EWS, acquired in negative ion mode (panel (**A**)) and their optimised derivates, OEWS, (panel (**B**)) from defatted sunflower by-product. The phenolic metabolites and the lipid compounds are written in red and blue, respectively. The absolute intensity (normalisation level, NL) of the most intense signal of each spectrum is also reported on the top right of each spectrum.

In the quest for a rapid and cost-effective tool for determining biochemical changes after valorisation of a sunflower oil by-product through an optimised treatment, the capabilities of DART-HRMS were investigated. The comparison of the chemical profiles of sunflower wastes, such as EWS and their optimised (OEWS) powders, are shown in Figure 1, where representative DART-HRMS spectra are reported.

In the spectra of Figure 1, the phenolic metabolites are written in red, while the lipids are written in blue. Note that the optimised samples (OEWS) are characterised by higher relative abundances of deprotonated phenolic compounds, such as protocatechuic acid (*m*/*z* 153.0190), vanillic acid (*m*/*z* 167.0351), the isobaric molecules syringic acid/caffeic acid (*m*/*z* 179.0341), hydroxy-caffeic acid (*m*/*z* 195.0299), the isobaric metabolite sinapaldehyde/caffeic acid ethyl ester (*m*/*z* 207.0663), and hydroxycinnamoyl-glyceric acid (*m*/*z* 233.0459), as well as by lower levels of deprotonated fatty acids (hydroxybutanoic acid of *m*/*z* 137.0235, linoleic acid of *m*/*z* 279.2329, oleic acid of *m*/*z* 281.2486, palmitic acid of *m*/*z* 255.2332, hydroxypalmitic acid of *m*/*z* 271.2282, hydroxylinoleic acid of *m*/*z* 295.2280 and oxohexadecanoic acid of *m*/*z* 269.2126). A zoomed DART-HRMS spectrum (*m*/*z* 190–300) range is included in the supporting information as Supplementary Material (Figure S1). The statistical significance of the different levels of metabolites, observed after valorisation of the defatted sunflower by-product (EWS vs. OEWS), was assessed by univariate analysis. 

### 2.2. Effect of the Optimisation on the Most Informative DART-HRMS Ions

**Figure 2:**
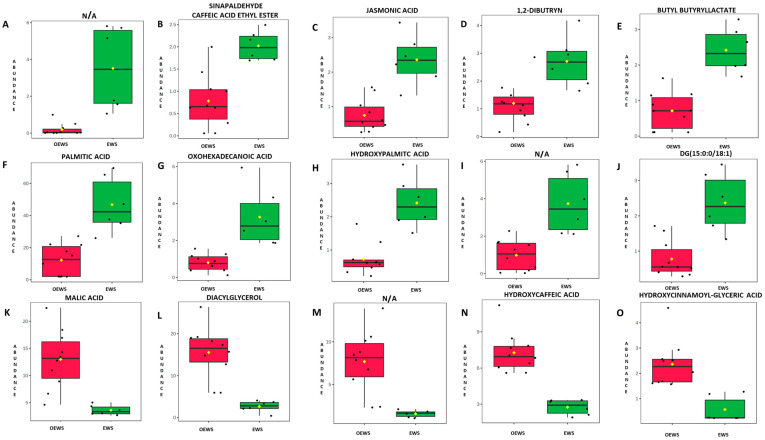
Box-whisker plots of the fifteen most statistically relevant (Padj ≤ 0.05) metabolites acquired by direct analysis in real-time high-resolution mass spectrometry (DART-HRMS). Each subfigure (from **A**–**O**) reports one of the metabolites with the tentatively assigned compound name reported on the top (N/A, not assigned). Green boxes represent the ethanol-wash solute (EWS) samples while red boxes represent the ultrasound-assisted optimised samples (OEWS). The bottom and top of each box represent the 25th and 75th percentiles, respectively; the mid-line indicates the 50th percentile or the median; the yellow square indicates the mean and the black circles represent the entire data range.

The combination of the t-test and fold change (FC) analyses provided the most significant metabolites of the EWS and OEWS fingerprintings with a Padj ≤ 0.05 and at least 2-FC (log_2_FC > 1 or log_2_FC < −1). The resulting volcano plot is shown in Figure S2. In Figure 2, the box-whisker plots of the fifteen most statistically significant metabolic ions are shown. As reported in Table 1 and Figure 2, the EWS matrix was characterised by a significantly higher relative abundance of lipid molecules, while the optimised EWS (OEWS) showed a higher incidence of the phenol hydroxycaffeic acid and phenolipid hydroxycinnamoyl-glyceric acid. By looking at the box-whisker plots (Figure 2), we can observe that the valorisation process led to a partial removal of the lipid fraction. Therefore, the univariate statistical analysis confirmed what could be already observed in the DART-HRMS spectra at first glance. Furthermore, the box-whisker plots revealed a wide variability of the relative abundance of malic acid, diacylglycerol and sinapaldehyde/caffeic acid ethyl ester within the defatted sunflower processed substrates (OEWS). This variability could be only partially explained by the lack of proper standardisation of the lab-made valorisation process. Despite the fact that sunflower cultivation from similar genotypes and agronomic management is highly standardised in terms of harvested seed quality traits across wide cropping areas, the seed’s chemical composition might change within the same batch due to the specific influence of cofounding factors operating at a micro–local level during the crop growing season, such as stress due to water scarcity, different nutrients, plant recovery patterns, as well as the occurring of diverse temporal phenology and ripening phases [32,33].

### 2.3. Multivariate Statistical Analysis of the DART-HRMS Fingerprintings

**Figure 3:**
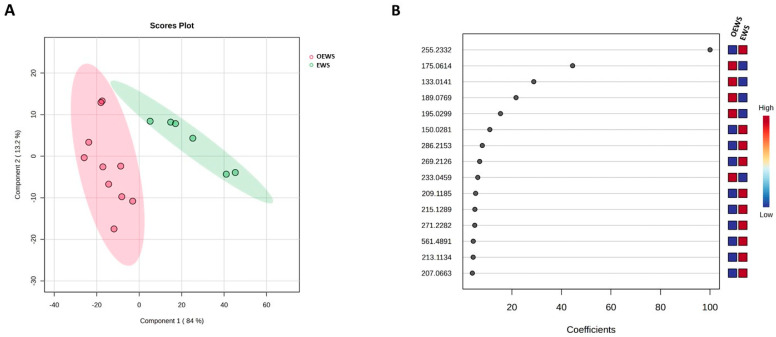
Outcomes of the partial least squared statistical analysis (PLS-DA) on direct analysis in real-time high-resolution mass spectrometry (DART-HRMS) data previously extracted by volcano plot. On the left, the PLS-DA scores plot (panel (**A**)) and, on the right, the PLS-DA coefficient plot of the 15 most informative ions (panel (**B**)). EWS, ethanol-wash solutes (green circles); OEWS, optimised ultrasound-assisted EWS (red circles). The putative assignments of the informative ions are reported in Table 1.

The first goal of the study was to assess the reliability of DART-HRMS fingerprinting as a rapid tool for the metabolomic assessment of the two investigated recycled defatted sunflower by-products resulting from the seed oil extraction. To this aim, the PLS-DA scores plot, generated with the most significant ions previously retrieved by univariate analysis, was built up and is reported in Figure 3A. The PLS-DA scores plot provides a graphical illustration of the potential of DART-HRMS for providing an accurate distinction between EWS and its optimised derivates (OEWS). Indeed, the PLS-DA allowed an accurate and well-defined spatial discrimination and clustering of samples from the EWS and OEWS sunflower processed by-products. In detail, the graphical space was defined by two first components C1 and C2, which explained 84.0% and 13.2% of the total variance of the model, respectively (Figure 3A). The high discrimination degree between the waste soluble extract (EWS) and the related optimised extract (OEWS) confirmed the differences in the metabolomic fingerprinting of the two sunflower matrices previously observed in the spectra, and it proved the DART-HRMS the ability to capture the chemical changes caused by the valorisation procedure. The PLS-DA coefficient plot graphically shows the coefficient values of the fifteen ions (*m*/*z* values) that contributed the most to the discrimination of the two groups (Figure 3B).

Table 1 reports the most significant (coefficient > 60) ions that characterised the chemical comparison between EWS and OEWS samples coming from the combination of ethanol-washing and ultrasound treatment optimisation process; the adopted statistical modelling reveals the presence of fatty acids of *m*/*z* 255.2332 (palmitic acid) and of *m*/*z* 271.2282 (hydroxypalmitic acid). Moreover, the most relevant metabolites of the OEWS samples are those of diacylglycerol (*m*/*z* 175.0614), malic acid (*m*/*z* 133.0141), hydroxycaffeic acid (*m*/*z* 195.0299), and hydroxycinnamoyl-(x)-glyceric acid (*m*/*z* 233.0459). These outcomes suggest that the physical treatment based on ultrasounds could be responsible for the removal of some fatty acids or other lipolytic compounds. The main difference in phenols composition between EWS and OEWS is the presence of a relatively high abundance of caffeic and cinnamic acid derivatives [hydroxycaffeic acid and the phenolipid hydroxycinnamoyl-(x)-glyceric acid] in OEWS. As shown in Figure 1, the other phenols were not removed by the valorisation process; thus, their relative abundance was mostly similar in EWS and OEWS, as confirmed by the absence of a statistically significant effect. This experimental finding suggests that the final step of the valorisation process applied to EWS, an ultrasound-assisted physical treatment, leads to a decrease in the lipid fraction with a consequent enrichment in the phenolic content. It is likely that the ultrasound treatment could promote the esterification of hydroxycinnamoyl compounds with glyceric acid [34]. Thus, considering the increased levels of phenolic compounds and the reduced levels of lipids, OEWS could be considered a feed and food additive to enhance storage stability [16]. This experimental outcome confirmed that the removal of lipids led to a highly purified content of phenolic compounds (i.e., mainly phenolic acids) in the OEWS defatted sunflower processed matrix, which allows us to state that the investigated OEWS might be considered a beneficial and healthy food ingredient thanks to its enhanced antioxidant activity. Moreover, this higher relative abundance of phenolic structured lipids having natural antioxidant properties that characterised the OEWS should be further exploited by using them as a supplement to improve preservation and to prolong the shelf-life of supplemented feed and food, especially high-fat foods since it might improve its oxidation stability, mostly for unsaturated long-chain fatty acids [35]. Indeed, the autoxidation of lipids is known to bring about deleterious effects and, therefore, ensuring a high quality of lipid-containing products and prolonging their storage time is directly dependent on the presence of antioxidants [36]. Therefore, the recovery of phenolic compounds from these defatted sunflower by-products could be of interest for food and/or nutraceutical industries due to their great potential as antioxidants [37]. For example, vanillic acid has been reported to exert multiple biological effects, such as activity against diseases like cancer, diabetes, obesity, and neurodegenerative, cardiovascular, and liver disorders [24]. Syringic acid is helpful as an antioxidant, anti-inflammatory, anti-diabetic, and hepatoprotective agent, and it also works as a barrier to lifestyle diseases [38]. Indeed, it is considered an excellent therapeutic agent in various diseases (diabetes, cancer, neuro and liver damages) and it possesses antimicrobial and anti-inflammatory properties [39]. It is also part of the subgroup of hydroxybenzoic acids, whose positive biological activities seem to be linked to the methoxy groups of the benzene ring [38]. Caffeic acid is produced via hydrolysis of the chlorogenic acid [40]. It has antioxidant properties that prevent the production of reactive oxygen species (ROS), which are associated with the development of several diseases. Protocatechuic acid is a powerful antioxidant with antibacterial, anticancer, antidiabetic, and anti-aging activities [41]. Hydroxycaffeic acid belongs to the class of hydroxycinnamic acids and it is known as a powerful antioxidant [42]. It is likely that the ultrasound treatment contributes to the esterification of hydroxycinnamoyl compounds with glyceric acid, producing hydroxycinnamoyl-(x)-glyceric acid, a phenolipid with amphiphilic properties. As suggested by the literature [43], this latter acid improves its solubility in less polar systems such as oils and fats.

While the enhancement of the phenolic fraction seemed to be efficient, some inter-batch differences could be observed, especially in terms of removal of fatty acids and their derivates (palmitic acid, hydroxy-palmitic acid, dibutryn and butyl butyryllactate) and of some organic acids (malic and jasmonic acids), as depicted by the box-whisker plots reported in Figure 2. As already stated above, the relatively high standard deviation detected for some of these significant ions could be related both to an intrinsic variability (e.g., cropping and harvesting influences) within the defatted sunflower by-products and to the lack of standardisation of the investigated optimisation process. Since, on the one hand, the presence of free fatty acids (i.e., oxohexadecanoic acid) can worsen the chance of rancidity, with only small amounts of them leading to a reduction of food quality [44], and, on the other hand, the increase of specific phenolipid compounds [i.e., hydroxycinnamoyl-(x)-glyceric acid] acts positively as a natural antioxidant for lipid food preservation, a more effective concentration of selected desirable compounds should be pursued. In this respect, a further challenge should be the improvement of some of the operative steps in the optimisation process, especially to increase the presence of polyphenols.

The findings of this study suggest that DART-HRMS provides a direct analysis of EWS and OEWS under ambient conditions for near-real-time analysis of the molecular content by way of a rapid and sustainable fingerprinting. Coupled with the statistical analysis method, this AMS technique strives to shorten the timescale of the analysis of agricultural byproducts, provides a more in-depth insight at molecular level comparing to conventional antioxidant analysis, and allows verifying the adequacy of the valorisation processes. The application of DART-HRMS for preliminary screening of valorised agricultural by-products has great potential to guide the optimisation of the recycling process and the choice of reference methods for the final chemical characterisation, thereby reducing costs of analysis.

## 3. Materials and Methods

### 3.1. Preparation of the Sunflower Waste Processed Samples and Experimental Dataset

The sunflower EWS were obtained from the sunflower meal following the steps reported previously by Gandova et al. [45]. Briefly, the sunflower meal was treated four times with an aqueous ethanol solution (75%), then the spent ethanol-washed liquids were collected, mixed, vacuum concentrated and lyophilised to recover the powdery EWS. Distilled water was used as a solvent for the extraction of EWS powder in a ratio of 1/50 (*w*/*v*), at room temperature for 20 min, under constant stirring (220 rpm). After that, the ultrasound assisted extraction (UAE) of this solution was performed with the SONOPULS ultrasonic homogeniser at a 20 ± 0.5 kHz frequency with the KE76 tip (BANDELIN electronic GmbH & Co. KG, Berlin, Germany). The independent variables studied were amplitude (10–35%) and time (1–8 min). After the first ultrasound treatment, the samples were centrifuged at 9500× *g*, at 4 °C for 5 min, then a second UAE was carried out under the same conditions. The optimised ultrasound extraction (OEWS) was carried out, taking into account the surface response methodology (SRM) previously applied for rapeseed EWS [14], and adapted to sunflower samples, with an optimised amplitude and time of 27% and 1.6 min, respectively. Three different batches of waste (EWS) were collected and processed (OEWS) in duplicate as described previously [16], for a final total amount of 3 batches of EWS and 6 resulting samples of OEWS. Both EWS and OEWS samples were further analysed by DART-HRMS according to the chemical stamp-based protocol for the optimisation of sunflower waste in an ultrasound-assisted processing procedure, thus ensuring a high level of compliance with reproducibility, interpretability, and reusability of mass spectrometry data. However, before DART-HRMS, one OEWS sample was removed from the dataset because of a failure during a handling pre-processing step, which could not be re-processed due to a limitation of the specific sunflower waste batch.

### 3.2. Fingerprinting Analysis by DART-HRMS

An amount of 1 g of each sample was extracted by 10 mL ethylacetate (Sigma Aldrich, Darmstadt, Germany). Non-targeted analyses of the extracts were carried out by DART SVP 100 source (IonSense, Saugus, MA, USA) coupled with an Exactive Plus Orbitrap Mass Spectrometer (Thermo Fisher Scientific, Waltham, MA, USA). A 5 µL volume of each extracted sample was spiked on a glass capillary rod that was then positioned onto a house-made holder of the Dip-it^®^ autosampler (IonSense, Saugus, MA, USA). To facilitate the formation of ammonium adducts, a small volume of NH_3_ (33% purity, from Sigma Aldrich, St. Louis, MO, USA) was positioned in front of the mass spectrometer inlet between the source and the autosampler. Subsequently, the melting point tubes were automatically moved by the autosampler at a constant speed of 0.3 mm/s through the DART gun exit and ceramic tube of the Vapur interface, in front of the orbitrap MS (Thermo Fisher Scientific, Waltham, MA, USA). The parameters of the DART and the Orbitrap analyser were set as described in previous studies [28,29]. The resolution was set to 70,000 FWHM (full width at half maximum) and the mass range was 75–1125 Da in negative ion mode. The extracts were analysed in duplicate and the spectra were converted into .csv files as already described in a previous study [29]. The tentative assignment of the ions was performed by interrogating the foodomics database library (FOODB, www.foodb.ca, accessed on 25 January 2024) and setting the volcano plotr threshold at ≤5 ppm. In order to confirm an ion assignment retrieved by the FOODB library, a literature search was also carried out to confirm their presence in the spectra.

### 3.3. Statistical Analysis

The DART-HRMS spectral data were pre-processed by using the R Statistical Software (release 4.3.2) with the MALDIquant package [46] and statistically analysed using the web platform 5.0 (www.metaboanalyst.ca, accessed on 25 January 2024). Firstly, the isotopes in the spectral data were removed using internally developed R codes. Specifically, the ions with a signal-to-ratio lower than 5 were removed, and the signals were aligned with a tolerance of 15 ppm. Afterwards, the absolute intensities of each spectrum were normalised by relative intensity of the most intense signal. To retrieve the most significant *m*/*z* values capable of differentiating the EWS and the related optimised samples (OEWS), a volcano plot was built to obtain the Padj-values resulting from the non-parametric t-test with Padj by false discovery rate (FDR). The volcano plot has -log10 on the y axis and fold-change (log_2_FC) on the x axis. Afterwards, the most significant molecular features were submitted to PLS-DA. The PLS-DA scores plot and coefficient plot with the most discriminant variables were visualised. The box-whisker plots of the 15 most significant variables with Padj ≤ 0.05 and at least 2-FC (log_2_FC > 1 or log_2_FC < −1) were generated.

## 4. Conclusions

In this study, an innovative approach where an ultrasound assisted extraction was used to optimise recycled defatted sunflower ethanol-wash solutes (EWS) was tested to assess whether this process significantly influenced the biochemical composition of the optimised derivate (OEWS). To this aim, the main outcomes highlighted the reliable performance of DART-HRMS used as an AMS technique for a rapid identification of metabolomic differences in the chemical fingerprinting between EWS and their optimised ultra-sound derivates. Thanks to the aid of a classification model with a PLS-DA classifier, the analytical and statistical approach provided an accurate and well-defined spatial discrimination and clustering of EWS and OEWS samples based on the identification of a restricted pool of 15 informative ions.

The experimental outcomes might provide valuable operative proposals for the valorisation of sunflower biowastes and by-products, especially within the zero-waste vision of the 2030 European Agenda. In fact, the present pilot lab-scale experimental work showcases the possibility to combine valorisation of food by-products processing for the recovery of antioxidants and bioactive compounds with the principles of green chemistry. As further steps to take, it is advisable to proceed with a more standardised processing of defatted sunflower by-products, together with a more selective concentration of desirable bioactive compounds.

Furthermore, our methodological and analytical approach could be used as an operative benchmark serving as a technical framework to be adopted for nutritional-wise recycling of other similar plant by-products. The application of these robust and easy-to-implement technological processes would be valuable for any company involved in crop waste recycling within a more sustainable and envisioned supply chain.

## Figures and Tables

**Table 1:** Discriminative compounds that enabled the differentiation between EWS and OEWS. Observed *m*/*z*, theoretical *m*/*z* mass, error (ppm), elemental formula, type of ion, log_2_ fold change (FC), adjusted *p*-value (Padj), and tentative assignment are listed.

*m*/*z*	*m*/*z* Theoretical Mass	Error (ppm)	Elemental Formula	Type of Ion	log_2_(FC)	Padj	Tentative Assignment
				EWS			
150.0281	−	−	C_8_H_8_O_2_	[M − H]^−^	−4.197	0.023	N/A
207.0663	207.0663	0	C_11_H_12_O_4_	[M − H]^−^	−1.368	0.046	Sinapaldehyde/Caffeic acid ethyl ester
209.1185	209.1183	0.95	C_12_H_18_O_3_	[M − H]^−^	−1.660	0.023	Jasmonic acid
213.1134	213.1127	−	C_11_H_20_O_5_	[M – H − H_2_O]^−^	−1.175	0.036	1,2-Dibutyrin
215.1289	215.1289	0	C_11_H_20_O_4_	[M − H]^−^	−1.760	0.023	Butyl butyryllactate
255.2332	255.233	0.78	C_16_H_32_O_2_	[M − H]^−^	−1.942	0.028	Palmitic acid
269.2126	269.2122	1.5	C_16_H_30_O_3_	[M − H]^−^	−2.029	0.015	Oxohexadecanoic acid
271.2282	271.2279	1.10	C_16_H_32_O_3_	[M − H]^−^	−1.741	0.022	Hydroxypalmitic acid
286.2153	−	−	C_7_H_6_O_3_	[M − H]^−^	−1.927	0.036	N/A
561.4891	561.4883	1.4	C_36_H_68_O_5_	[M – H − H_2_O]^−^	−1.605	0.023	DG (15:0/0:0/18:1)
				OEWS			
133.0141	133.0142	−0.7	C_4_H_6_O_5_	[M − H]^−^	1.844	0.022	Malic acid
175.0614	175.0612	1.14	C_7_H_12_O_5_	[M − H]^−^	2.546	0.015	Diacylglycerol
189.0769	189.0768	0.5	C_8_H_14_O_5_	[M − H]^−^	2.298	0.015	N/A
195.0299	195.0299	0	C_9_H_8_O_5_	[M − H]^−^	1.405	0.015	Hydroxycaffeic acid
233.0459	233.0450	3.8	C_12_H_10_O_5_	[M – H − H_2_O]^−^	2.064	0.023	Hydroxycinnamoyl-(x)-glyceric acid

EWS, ethanol-wash solutes; OEWS, optimised ultrasound-assisted EWS; N/A, not assigned. DG, diacylglycerol.

## Data Availability

Research data will be provided upon request.

## Supplementary Materials

The following supporting information can be downloaded at: https://www.mdpi.com/article/10.3390/molecules29174092/s1, Figure S1: Zoomed DART-HRMS spectrum (*m*/*z* 190–300) range. Figure S2: Volcano plot.
