# Supplementary material for: Rapid Assessment of Metabolomic Fingerprinting of Recycled Sunflower By-Products via DART-HRMS
Source: Molecules. 2024 Aug 29;29(17):4092. doi: 10.3390/molecules29174092 (PMC11397051; doi:10.3390/molecules29174092)
Supplement: Supplementary file 1 [file molecules-29-04092-s001.zip › molecules-3153641-supplementary.pdf]

Supplementary materials

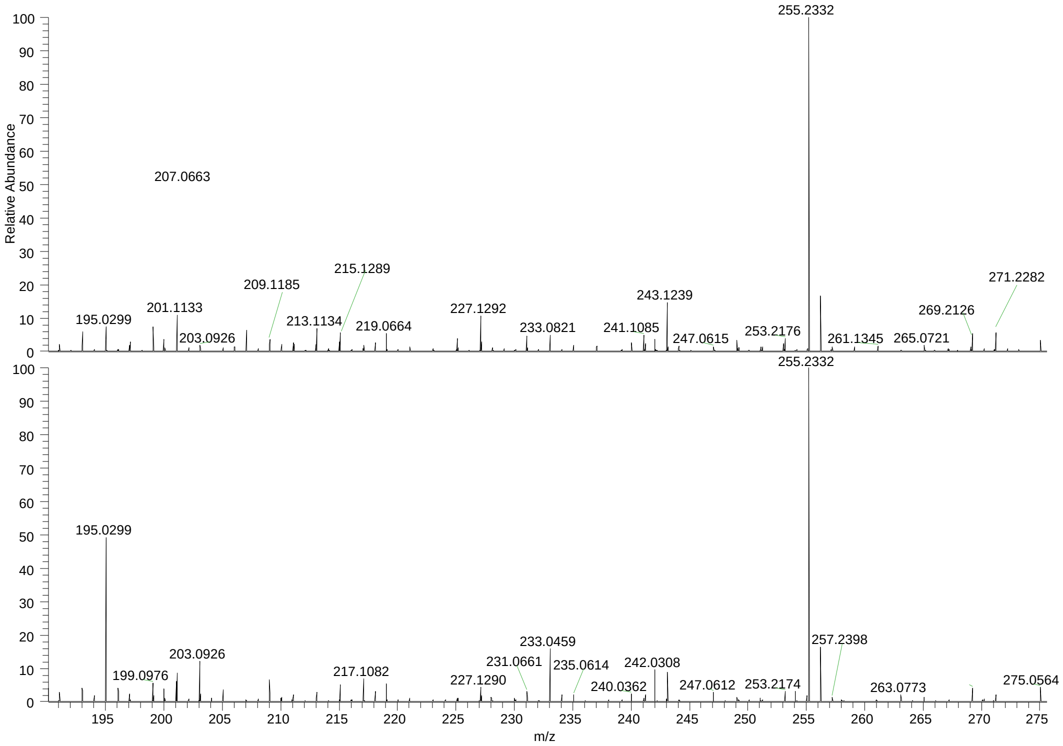

Figure S1. Zoomed DART-HRMS spectrum (*m*/*z* 190-300) range.

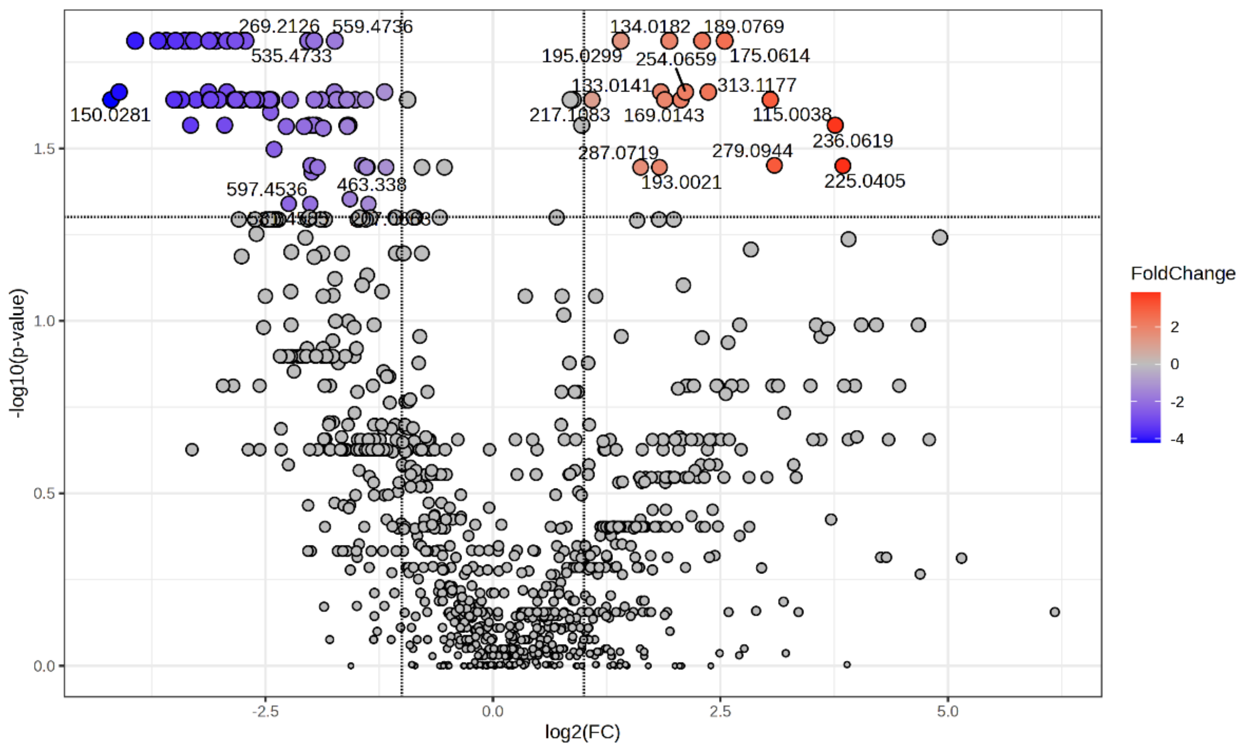

Figure S2. Volcano plot.

A volcano plot was built to visualize the P-values resulting from the nonparametric *t*-test with P-value adjusted (Padj) by false discovery rate (FDR) (–log10pvalue) and fold-change (log2FC). The ions with a Padj ≤ 0.05 and at least a 2-fold change (log2FC > 1 or log2FC < -1) were considered as statistically significant.
